# Supplementary material for: Empirical methods for controlling false positives and estimating confidence in ChIP-Seq peaks
Source: BMC Bioinformatics. 2008 Dec 5;9:523. doi: 10.1186/1471-2105-9-523 (PMC2628906; doi:10.1186/1471-2105-9-523)
Supplement: Additional File 1 — A variety of html documents from the USeq web site detailing the available applications, their best usage, output file type descriptions, command line menus, etc. [file 1471-2105-9-523-S1.zip › USeqUserGuides/JavaDoc.zip!/JavaDoc/util/bio/cluster/class-use/Cluster.html]

Uses of Class util.bio.cluster.Cluster


|  |  |  |
| --- | --- | --- |
| **Overview** \| **Package** \| **Class** \| **Use** \| **Tree** \| **Deprecated** \| **Index** \| **Help** | |  |
| PREV   NEXT | **FRAMES**    **NO FRAMES**     **All Classes** |  |


---


## **Uses of Class util.bio.cluster.Cluster**

| Packages that use Cluster | |
| --- | --- |
| **util.bio.cluster** | Clustering apps and methods. |

Uses of Cluster in util.bio.cluster

| Methods in util.bio.cluster that return Cluster | |
| --- | --- |
| `Cluster` | `Cluster.getBottomCluster()` |
| `Cluster` | `HierarchicalClustering.getMegaCluster()` |
| `Cluster` | `Cluster.getParentCluster()` |
| `Cluster` | `Cluster.getTopCluster()` |

| Methods in util.bio.cluster with parameters of type Cluster | |
| --- | --- |
| `double` | `ClusterDrawPanel.fetchBottomCenterY(Cluster cluster)`             Recurse through clusters always going lower to find center |
| `double` | `ClusterDrawPanel.fetchClusterCenterY(Cluster c)`             Returns the 'middle' Y coordinate for the cluster. |
| `double` | `ClusterDrawPanel.fetchTopCenterY(Cluster cluster)`             Recurse through clusters always going higher to find center |
| `void` | `Cluster.setBottomCluster(Cluster bottomCluster)` |
| `void` | `Cluster.setParentCluster(Cluster parentCluster)` |
| `void` | `Cluster.setTopCluster(Cluster topCluster)` |

Constructors in util.bio.cluster with parameters of type Cluster

`Cluster(Cluster one,
Cluster two,
double correlationCoefficient,
java.lang.StringBuffer results)`
  
          Secondary Constructor

---


|  |  |  |
| --- | --- | --- |
| **Overview** \| **Package** \| **Class** \| **Use** \| **Tree** \| **Deprecated** \| **Index** \| **Help** | |  |
| PREV   NEXT | **FRAMES**    **NO FRAMES**     **All Classes** |  |


---
